# A Novel Clinical Score Predicting the Presence of Fatty Pancreas

**DOI:** 10.3390/jcm10245843

**Published:** 2021-12-13

**Authors:** Tawfik Khoury, Amir Mari, Wisam Sbeit

**Affiliations:** 1 Department of Gastroenterology, Galilee Medical Center, Nahariya 22100, Israel; wisams@gmc.gov.il; 2 Faculty of Medicine in the Galilee, Bar-Ilan University, Safed 5290002, Israel; amir_mari@nazhosp.com; 3 Gastroenterology and Endoscopy Units, The Nazareth Hospital, EMMS, Nazareth 16100, Israel

**Keywords:** pancreas, fat, score, prediction, clinical, parameters

## Abstract

Background: Fatty pancreas (FP) has become an increasingly encountered entity in recent years. Several studies have shown an association with several disease states. Aims: we aimed to generate a simple non-invasive scoring model to predict the presence of FP. Method: We performed a retrospective cross-sectional analysis at Galilee Medical Center. Inclusion criteria included patients who underwent endoscopic ultrasound (EUS) for hepatobiliary indications and who had either hyperechogenic pancreas consistent with FP or no sonographic evidence of fatty pancreas. Results: We included 569 patients. Among them, 78 patients had FP by EUS and 491 patients did not have FP. On univariate analysis, obesity (odds ratio (OR) 5.11, *p* < 0.0001), hyperlipidemia (OR 2.86, *p* = 0.0005), smoking (OR 2.02, *p* = 0.04), hypertension (OR 2.58, *p* = 0.0001) and fatty liver (OR 5.94, *p* < 0.0001) were predictive of FP. On multivariate analysis, obesity (OR 4.02, *p* < 0.0001), hyperlipidemia (OR 2.22, *p* = 0.01) and fatty liver (OR 4.80, *p* < 0.0001) remained significantly associated with FP. We developed a diagnostic score which included three parameters that were significant on multivariate regression analysis, with assignment of weights for each variable according to the OR estimate. A low cut-off score of ≤1 was associated with a negative predictive value (NPV) of 98.1% for FP, whereas a high cut-off score of ≥2 was associated with a positive predictive value (PPV) of 35–56%. Conclusion: We recommend incorporating this simple score as an aid to identify individuals with FP.

## 1. Introduction

Fatty pancreas (FP) is characterized by fat deposition within the pancreatic parenchyma [1], with a prevalence ranging from 16–35% according to abdominal imaging [2,3]. FP is an increasingly encountered incidental finding on abdominal imaging including endoscopic ultrasound (EUS) [4,5]. While the first observation of FP was reported as early as the 1930s, the clinical implications of this finding were not deeply investigated for several decades, as most clinicians perceived this condition as a benign condition without any future clinical consequences. In recent years, FP was demonstrated to be associated with several comorbid diseases [6,7,8,9]. To date, studies that addressed this coexistence reported only disease states that were shown to be associated with FP, such as diabetes mellitus (DM) [10,11,12], metabolic syndrome [6,13], cardiovascular disease [14] and fatty liver [15]. Recent data have revealed a more severe associated comorbid diseases. Previous studies showed a significant association of pancreatic intra-epithelial neoplasia with pancreatic fat accumulation (OR of 6.1; *p* = 0.001) [7] and FP (OR 17.86; 95% CI 4.935–88.12) [16]. Additionally, FP was shown to promote the malignant potential and spreading of tumors [17]. Notably, recently published data demonstrated that FP was associated with pre-cancerous main duct intraductal papillary mucinous neoplasm (M-IPMN) (OR 2.69, 95% CI 1.05–6.9, *p* = 0.04) [18] and represented a risk factor for acute pancreatitis [1]. All of these studies reported sole association with FP.

However, none have tried to develop a clinical score encompassing the most significant predictive factors for the presence of FP. Therefore, the aim of the present study is to develop a clinical score based on clinical data that predicts the presence of FP.

## 2. Study Design

A retrospective data analysis of all patients who underwent EUS for hepatobiliary indication at Galilee Medical Center from 1 January 2015 to 1 January 2020 was conducted. Extracted data included demographics (age and gender) and background diseases. The study was approved by the local institutional ethics committee. Written informed consent was waived due to the retrospective non-interventional study design.

## 3. Methods

All EUS examinations were performed by an experienced advanced endoscopist and were reviewed and approved by another EUS expert. Patients were sedated with intravenous midazolam and propofol according to the decision of the endoscopist.

### 3.1. Study Definitions

Obesity was defined as having a body mass index (BMI) > 30 kg/m^2^. Cirrhosis was defined as end-stage liver disease accompanied by symptoms related to cirrhosis and synthetic function impairment. Obstructive lung disease was defined as a respiratory disease characterized by airway obstruction and demonstrated by reduced FEV1/FVC < 0.7 using spirometry. Congestive heart failure was defined as a reduced heart pump function diagnosed by echocardiogram. Ischemic heart disease was defined by typical symptoms of angina pectoris coupled with atherosclerotic coronary arteries. Diabetes mellitus was defined as having both typical symptoms including polydipsia and polyuria coupled with hemoglobin A1c level ≥ 6.5%. Hypertension was defined as having systolic blood pressure at ≥140 mm Hg and/or diastolic blood pressure at ≥90 mm Hg following 2–3 visits at 1–4-week intervals. Hyperlipidemia was defined as having a serum cholesterol level of ≥240 mg/dl. Fatty liver was diagnosed as having a hyperechogenic texture in the same EUS exam and in the absence of significant alcohol consumption.

### 3.2. Fatty Pancreas Diagnosis in Our Cohort

The diagnosis of FP in our cohort was performed by EUS examinations as demonstrated by the hyperechogenicity of the pancreatic parenchyma compared to the kidneys or the spleen [19] using linear echoendoscope (Pentax, Tokyo, Japan), model 3870.

## 4. Statistical Analysis

The main end point of our study was to predict the presence or absence of FP by a combination of simple clinically relevant parameters. A univariate descriptive statistic was used to compare patients with and without FP. Data was reported as frequencies (percentages) for non-continuous categorical variables and as mean ± standard deviation for quantitative continuous variables. Univariate and multivariate logistic regression models were used to evaluate the effect of baseline parameters on the presence of FP by reporting odds ratios (OR) and confidence intervals (CI). Finally, we generated a score predicting FP by attributing weights to the parameters that were significant on multivariate analysis according to the OR estimates. The overall diagnostic accuracy of the scoring system was determined by a receiver operating characteristics (ROC) curve. A threshold for statistical significance was set at a *p* value < 0.05. All analyses were performed by an experienced statistician using statistical analysis software (SAS vs. 9.4 Copyright (c) 2016 by SAS Institute Inc., Cary, NC, USA).

## 5. Results

### 5.1. Demographics and Baseline Characteristics

Overall, we included 569 patients. The indications of the EUS examinations were as follows: abdominal pain with liver enzyme abnormality (374 patients), investigation for acute pancreatitis episodes with unknown etiology (84 patients) and suspected pancreato-biliary malignancies (111 patients). Among them, 78 patients had FP by EUS (group A), as compared to 491 patients who did not have FP (group B). The average age in group A was 62.1 ± 14.1 vs. 62.9 ± 14.1 years in group B. The prevalence of obesity, hyperlipidemia, hypertension and fatty liver were higher in group A (53.8%, 24.4%, 48.7% and 84.6%, respectively) compared to group B (19.3%, 10.2%, 26.9% and 47.2%, respectively). Notably, there was no difference in the gender predominance between the two groups. Table 1 demonstrates the demographics and the clinical characteristics of the study cohort. Notably, there was no difference in the rate of side branch intraductal main papillary mucinous neoplasm (IPMN) (16.7% in group A vs. 20.6% in group B, *p* = 0.2) or for mixed type IPMN (2.6% in group A vs. 2.7% in group B, *p* = 0.4). Main duct IPMN was commoner in group A (10.3%), compared to 3.3% in group B (*p* = 0.02). Moreover, there was no difference in the rate of pancreatic adenocarcinoma or neuroendocrine tumor in group A (12.8% and 5.1%, respectively) vs. 8.2% and 5.9% in group B (*p* = 0.09 and 0.4), respectively.

### 5.2. Univariate Analysis of Parameters Associated with the Presence of Fatty Pancreas

On univariate analysis, several parameters were associated with the presence of FP, including obesity (OR 5.11, 95% CI 3.09–8.46, *p* < 0.0001), hyperlipidemia (OR 2.86, 95% CI 1.58–5.18, *p* = 0.0005), smoking (OR 2.02, 95% CI 1.04–3.93, *p* = 0.04), hypertension (OR 2.58, 95% CI 1.59–4.19, *p* = 0.0001) and fatty liver (OR 5.94, 95% CI 3.16–11.16, *p* < 0.0001), while there was a trend for association with diabetes mellitus (OR 1.63, 95% CI 0.95–2.79, *p* = 0.07). Notably, there was no effect of age and gender on the presence of FP (Table 2). On multivariate logistic regression analysis, 3 parameters remained significantly associated with the presence of FP, including obesity (OR 4.02, 95% CI 2.38–6.80, *p* < 0.0001), hyperlipidemia (OR 2.22, 95% CI 1.17–4.23, *p* = 0.01) and fatty liver (OR 4.80, 95% CI 2.48–9.27, *p* < 0.0001).

## 6. Model Building

**Figure 1:**
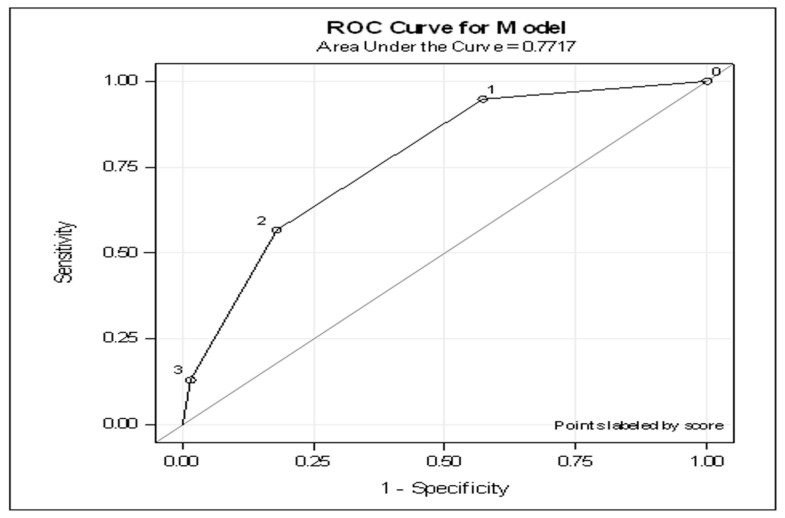
Demonstrates the ROC curve analysis of the score including obesity, hyperlipidemia and fatty liver.

For the generation of a diagnostic score, we assigned weights for each variable according to the OR estimates in the multiple logistic regression model. Accordingly, obesity, hyperlipidemia and fatty liver received 1 point each, making a maximal score of 3 points. The ROC for this score is 0.77 (OR 3.54, 95% CI 2.56–4.89, *p* < 0.0001) (Figure 1). Further analysis to assess the diagnostic performance of the score in terms of sensitivity and specificity, positive predictive value (PPV) and negative predictive value (NPV) revealed that a score of ≥2 was associated with a very high specificity and variable range of sensitivity, while a score of ≤ 1 was associated with a high sensitivity and excellent NPV (Table 3).

## 7. Discussion

Although FP was shown previously to be an incidental and benign finding encountered on abdominal imaging studies [20], the clinical consequences of this condition have rarely been investigated and discussed among clinicians as its diagnosis was not routinely translated into clinical decision-making. To date, from the studies that have reported several disease associations with FP, none have tried to incorporate clinical parameters into a score that predicts the presence of FP. Herein, we generated a simple, easily available score with high accuracy based on the presence of three variables, including: obesity, hyperlipidemia and fatty liver with a good ROC of 0.77 and a very high specificity of 82.1–98.4%. Among patients with a score of 1, FP was absent in 98.1%, while among patients with a score of ≥2, FP was present in 35–56%.In fact, besides the incidental identification of patients with FP, this score enables clinicians to proactively identify and diagnose patients with FP, especially in the current era where data that report an association of FP with severe and life-threatening diseases are accumulating as demonstrated by recent studies [1,18]. In particular, this refers to the association of FP with pancreatic adenocarcinoma [20] and main-duct IPMN [18]. To summarize, this was the first time to show that incorporating several predictors of fatty pancreas in one score improves the probability of identifying this disorder. Similarly, incorporating multiple predictors in a score was shown to improve the probability of predicting common bile duct stone [21]. Therefore, our developed score built from modifiable variables opens the door to developing further accurate scores for FP in order to optimize the identification of patients with FP and to apply appropriate intervention.

Therefore, this work provides clinicians with a simple score for identifying FP presence, as its identification is of paramount importance for assignment of treatment and follow-up plan for this potentially reversible disorder.

The limitations of this study its single-center nature and retrospective design and that we don’t have histological confirmation of pancreatic fatty infiltration; however, the straightforward endoscopic ultra-sonographic diagnosis of fatty pancreas as demonstrated by hyperechogenicity makes our diagnosis of FP reliable.

In conclusion, we have developed a scoring system for predicting the presence of FP. We recommend incorporating this score into clinical decision-making regarding the identification of such patients, especially as emerging evidence correlates FP to several pancreatic and extra-pancreatic diseases. Additional prospective studies should be performed to validate our findings and to better define the long-term consequences of FP.

## Figures and Tables

**Table 1:** Demographics and baseline characteristics.

	Group A (with FP)	Group B (without FP)
Number of patients	78	491
Age (years), mean ± SD	62.1 ± 14.1	62.9 ± 14.1
Gender, *N* (%)		
Male	41 (52.6)	261 (53.2)
Female	37 (47.4)	230 (46.8)
Obesity, *N* (%)	42 (53.8)	95 (19.3)
Cirrhosis, *N* (%)	0	5 (1)
Obstructive lung disease, *N* (%)	6 (7.7)	20 (4.1)
Congestive heart failure, *N* (%)	8 (10.3)	29 (5.9)
Hypothyroidism, *N* (%)	6 (7.7)	24 (4.9)
Hyperlipidemia, *N* (%)	19 (24.4)	50 (10.2)
Lipid profile		
Cholesterol level (mg/dL)	284	236
Triglycerides level (mg/dL)	263	162
Ischemic heart disease, *N* (%)	10 (12.8)	45 (9.2)
Smoking, *N* (%)	13 (16.7)	45 (9.2)
Diabetes mellitus, *N* (%)	22 (28.2)	96 (19.6)
Hypertension, *N* (%)	38 (48.7)	132 (26.9)
Fatty liver, *N* (%)	66 (84.6)	232 (47.2)

**Table 2:** Univariate analysis of parameters associated with fatty pancreas.

	Odds Ratio	95% CI	*p* Value
Age	0.99	0.98–1.01	0.58
Male gender	0.97	0.61–1.57	0.92
Obesity	5.11	3.09–8.46	<0.0001
Cirrhosis	0.56	0.02–13.55	0.72
Obstructive lung disease	2.06	0.81–5.24	0.13
Congestive heart failure	1.89	0.84–4.26	0.12
Hypothyroidism	1.71	0.69–4.26	0.25
Hyperlipidemia	2.86	1.58–5.18	0.0005
Ischemic heart disease	1.50	0.73–3.10	0.27
Smoking	2.02	1.04–3.93	0.04
Diabetes mellitus	1.63	0.95–2.79	0.07
Hypertension	2.58	1.59–4.19	0.0001
Fatty liver	5.94	3.16–11.16	<0.0001

**Table 3:** Predictive value of the scoring system.

	Score	
Score performance	≤1	≥2
Sensitivity	94.7–100%	13.2–56.6
Specificity	42.8%	82.1–98.4
Positive predictive value	13.4–20.3%	35–56%
Negative predictive value	98.1%	88–92.4
Likelihood ratio [+]	1–1.66	3.16–8.1
Likelihood ratio [–]	0.12	0.53–0.88
Interpretation	Absence of FP [98.1% certainty]	Presence of FP [35–56% certainty]

## Data Availability

The data are available with the corresponding authors at the Gastroenterology department at Galilee Medical Center and will be available upon reasonable request.

## Abbreviations

FP	fatty pancreas
EUS	endoscopic ultrasound
OR	odds ratio
NPV	negative predictive value
PPV	positive predictive value
CI	confidence interval
ROC	receiver operating characteristics
IPMN	intraductal main papillary mucinous neoplasm
